# A Multinomial Logistic Regression Analysis of Fertility Decisions and Associated Determinants Among Reproductive Age Women in Somaliland: Utilization of 2020 SLHDS Data

**DOI:** 10.1177/26884844251365809

**Published:** 2025-08-06

**Authors:** Hodo Abdikarim, Hibo Abdirashid, Mohamed A. Hussein, Abdirashid M. Yousuf, Abdisalam Hassan Muse, Saralees Nadarajah

**Affiliations:** ^1^ Faculty of Science and Humanities, School of Postgraduate Studies and Research (SPGSR), Amoud University, Borama, Somaliland.; ^2^ Research and Innovation Center, Amoud University, Borama, Somaliland.; ^3^ Department of Mathematics, University of Manchester, Manchester, UK.

**Keywords:** fertility decision, women, multinomial analysis, SLHDS data, Somaliland

## Abstract

**Background:**

Somaliland, despite a global trend toward lower fertility rates, exhibits a high total fertility rate of 5.7 per woman. This study aimed to investigate the prevalence and associated determinants of fertility decisions among married reproductive-age women in Somaliland.

**Methodology:**

A cross-sectional study using data from the 2020 Somaliland Health and Demographic Survey was conducted. A multinomial logistic regression analysis was employed to explore the association between individual-level (age, education, occupation, contraceptive use) and community-level (residence, region) factors and fertility decisions.

**Results:**

The study found that 54.4% of married reproductive-age women in Somaliland desire more children within the next 2 years, indicating a strong preference for larger families. Younger women, those residing in rural areas, and those with lower education levels were more likely to desire more children. Exposure to mass media was associated with a lower desire for more children, while contraceptive use intentions also significantly influenced fertility decisions.

**Conclusion:**

This study provides valuable insights into the complex interplay of socioeconomic, cultural, and individual factors shaping fertility decisions in Somaliland. The findings highlight the need for targeted interventions, including education, access to family planning services, and mass media campaigns, to empower women to make informed choices about family size.

## Introduction

Globally, many families are choosing to have fewer children, often prioritizing the well-being and opportunities they can provide for each child.^1^ This shift aligns with the “quantity–quality model,” which posits a trade-off between the number of children and the resources invested in each child’s development; as the cost of ensuring a higher quality of life for each child increases, families may opt for smaller families.^2^ Bloom et al. (2001) noted that the majority of countries are projected to experience low fertility rates in the coming decades.^3^ While not necessarily a crisis, this trend is becoming increasingly apparent, particularly in industrialized nations.^4^ Chand and Tung (2014) highlighted that declining fertility rates slow global population growth, but the primary concern is the acceleration of population aging and its potential impact on national economies and social structures.^5^

Research on fertility decisions has explored various modeling approaches. For example, Hondroyiannis (2004) used count models and normal distributions to simulate the number of live births in Greece, examining the influence of factors such as maternal age, household income, and education.^6^ This study found an inverse relationship between household income and women’s education levels and fertility decisions, suggesting that higher-income households may prioritize child quality over quantity. Amara (2015) found that regional unemployment rates and the availability of women’s health clinics had an inverse relationship with the number of children, while contextual factors like poverty rate in the area had a positive relationship with fertility in Tunisia.^7^ In this context, “inverse relationship” means that an increase in the factor (*e.g.,* income) is associated with a decrease in the number of children desired, while “positive relationship” means an increase in the factor (*e.g.,* poverty) is associated with an increase in the number of children desired.

The United Nations report estimates that between 2020 and 2100, there may be between 7.8 billion and 10.9 billion people on the planet.^8^ The world’s population grows by over 78 million people per year,^9^ and sub-Saharan Africa (SSA) is home to more than half of the world’s fertility, with an estimated 4.8 fertility rate,^10,11^ and Somaliland’s total fertility is higher, which is 5.7 per woman.^12^ Studies showed that a 40% rise in the present population would have a significant impact on economies, food production, general environment, and the global climate.^13–15^ Growing populations place additional demand on resources that are already under stress, which makes sustainable development extremely difficult. More than half of the expected growth in world population by 2050 is expected to come from Africa. With a predicted increase in population of over one billion, SSA countries will be responsible for almost half of global population growth between 2019 and 2050.^16^ The usage of contraceptives is necessary to achieve desired fertility and spacing between pregnancies.^17^ Furthermore, over half of women in several SSA nations who have more children still wish to have more,^18^ while Somaliland married women’s prevalence of contraceptives is low, which is only 7%.^12^

Moreover, numerous studies have found links between fertility decisions and factors at the individual and community levels, including age, married status, income, education, parity, and place of residence. Furthermore, a wealth of empirical data has demonstrated that fertility decisions differ greatly between nations.^19–23^ Understanding fertility decision’s magnitude has practical consequences for creating family planning programs and, more broadly, for reaching the sustainable development goal (SDG).^24–26^ Fertility decision is one of the determinants of contraceptive behavior and reproductive-related outcomes.^27^ Evaluating fertility desire is a crucial issue that needs to be taken into account, and many parts of both national and international planning depend on knowing the causes of the country’s unusual population increase. It can provide information to local governors and health care professionals to enhance supplementary health services.^28^

Therefore, the purpose of this study was to evaluate Somaliland’s fertility decisions and related determinants despite the country’s 5.7 overall fertility rate and 7% contraceptive use. Using 2020 SLHDS Data, which is the first-ever national representative demographic health survey, this study sought to investigate the prevalence and related factors of fertility decision among married reproductive age women in Somaliland.

## Materials and Methods

### Study area

This study was undertaken in Somaliland, a region located in East Africa. Geographically, Somaliland is bordered by Djibouti to the northwest, Ethiopia to the southwest, Somalia to the east, and the Gulf of Aden to the north. The total area of Somaliland is 176,119.2 km^2^, characterized by a mix of wet and dry climate conditions. The region is divided into six geopolitical areas: Awdal, Marodijeh, Sahil, Togdheer, Sanaag, and Sool. The population is estimated to be around 4.2 million, predominantly consisting of Somali ethnic groups who are primarily Muslim. Despite some progress in economic growth since declaring independence, Somaliland has faced challenges in achieving significant economic advancement. The lack of international recognition as an independent nation has restricted both foreign investments and international assistance. A significant portion of the population, in both rural and urban areas, depends on livestock products for their livelihood.

### Study design and setting

This study employs a cross-sectional design using data from the Somaliland Health and Demographic Survey (SLHDS) 2020. The study setting is Somaliland, a self-declared independent state in the Horn of Africa.

### Sample size and sampling process of Somaliland demographic and health survey

After cleaning and preprocessing, the study utilized 2857 sample of women between the ages of 15 and 49, which was collected from the SLHDS data. Sampling took into account the residency (urban, rural, and nomadic) and five geographic zones for stratification. Geographic information system software was used to choose the enumeration area (EA) for both urban and rural areas. The sampling frame consisted of 2806 residential structures, with a total of 1869 in urban areas and 937 in rural areas. A probability-based method was used to choose 35 EAs based on the size of the residential structures. Following the listing of households in 35 EAs, 10 primary sampling units (PSUs) were chosen using the probability proportional sampling technique from among the 35 EAs.^24^ to build a sample frame for temporary nomadic settlements (TNSs) inhabited by nomadic residents were applied. The projected number of households in each TNS served as a measure of size, and the list of TNSs was used as a sampling frame. There were 1448 TNS residential structures found, and both urban and rural inhabitants’ EAs were chosen in the same manner. Systematic sampling approaches were used to choose households as the final sampling unit.^29^

### Study variables

#### Outcome variable

This study investigated the fertility choices of married women who are of reproductive age. Fertility decisions were explicitly documented in the DHS data, which identified four different categories: “wants within 2 years,” “wants after 2 years,” “undecided,” and “wants no more.” As a result, the outcome variable is characterized as a multinomial outcome.

#### Independent variables

Previous research has highlighted several factors influencing fertility decisions in SSA.^27,30–32^ Consequently, this study categorizes potential correlations of fertility decisions into two main groups: individual-level and community-level variables. The individual-level factors include maternal age in 5-year groups, maternal education level, maternal occupation over the past year, husband attends school, husband works, contraceptive use and intentions, exposure to mass media, total number of children ever born, wealth index, sex of the household head, and number of living children in a household. Additionally, the study examines community-level factors, which encompass residence and region.

### Data source

The SLHDS served as the source of data for this study. Data collection was conducted by trained interviewers using the CSPro Android platform in both urban and rural environments during the survey. In each of the 10 enumeration regions within the geographic strata, 30 households were sampled. Similarly, 30 randomly selected households were surveyed in each enumeration region designated as nomadic areas. To maintain the accuracy and comprehensiveness of the household list, it was validated one day prior to the data collection in each TNS.

### Statistical analysis

The data were obtained from the SLHDS and subsequently underwent a cleaning process. Participants lacking outcome variables in the datasets were excluded prior to analysis. The data were then exported and analyzed using STATA version 17.0 software. Descriptive statistics, including mean, frequency, and percentage, were calculated. To evaluate the factors associated with fertility decisions, both statistical tests, including the chi-square test and regression analyses, were conducted.

We used multivariable multinomial logistic regression to examine the association between various factors and the outcome variable. Results are presented as relative risk ratios (RRRs) with 95% confidence intervals (CIs). The RRRs represent the relative change in the likelihood of belonging to one outcome category compared to the reference category, given a one-unit change in the predictor variable. An RRR > 1 indicates an increased likelihood, while an RRR < 1 indicates a decreased likelihood. Statistical significance was determined using a *p* value threshold of 0.05. Tables 1 and 2 have statistical significance markers for each parameter.

**Table 1.** Bivariate Logistic Results About Fertility Desire Among Reproductive Age Women in Somaliland

Background characteristics	Desire for more children				χ^2^ *p**-**value
	Wants within 2 years	Wants after 2 years	Undecided	Wants no more	
Maternal age in 5-year groups					
15–19	5 (45.45)	0 (0.00)	2 (18.18)	4 (36.36)	647.9734*
20–24	93 (93.00)	4 (4.00)	2 (2.00)	1 (1.00)	
25–29	283 (80.17)	5 (1.42)	31 (8.78)	34 (9.63)	
30–34	409 (70.76)	32 (5.54)	71 (12.28)	66 (11.42)	
35–39	474 (59.18)	47 (5.87)	122 (15.23)	158 (19.73)	
40–44	210 (33.87)	0 (0.00)	166 (26.77)	244 (39.35)	
45–49	82 (20.81)	12 (3.05)	86 (21.83)	214 (54.31)	
Region					
Awdal	145 (48.82)	2 (0.67)	38 (12.79)	112 (37.71)	193.8036*
Woqooyi Galbeed	363 (55.85)	30 (4.62)	71 (10.92)	186 (28.62)	
Togdheer	274 (47.90)	24 (4.20)	69 (12.06)	205 (35.84)	
Sool	471 (61.01)	8 (1.04)	184 (23.83)	109 (14.12)	
Sanaag	303 (53.53)	36 (6.36)	118 (20.85)	109 (19.26)	
Residence					
Rural	507 (50.15)	16 (1.58)	201 (19.88)	287 (28.39)	70.7427*
Urban	523 (52.09)	56 (5.58)	144 (14.34)	281 (27.99)	
Nomadic	526 (62.47)	28 (3.33)	135 (16.03)	153 (18.17)	
Maternal education level					
No Education	1,428 (55.82)	83 (3.24)	442 (17.28)	605 (23.65)	102.4772*
Primary	87 (41.63)	6 (2.87)	29 (13.88)	87 (41.63)	
Secondary	36 (47.37)	11 (14.47)	0 (0.00)	29 (38.16)	
Higher	5 (35.71)	0 (0.00)	9 (64.29)	0 (0.00)	
Sex of household head					
Male	1,016 (56.48)	67 (3.72)	296 (16.45)	420 (23.35)	11.5366*
Female	540 (51.04)	33 (3.12)	184 (17.39)	301 (28.45)	
Wealth Index					
Lowest	589 (53.35)	23 (2.08)	231 (20.92)	261 (23.64)	62.0740*
Second	217 (60.96)	16 (4.49)	37 (10.39)	86 (24.16)	
Middle	270 (57.57)	12 (2.56)	73 (15.57)	114 (24.31)	
Fourth	193 (49.87)	29 (7.49)	48 (12.40)	117 (30.23)	
Highest	287 (53.05)	20 (3.70)	91 (16.82)	143 (26.43)	
Total number of children ever born					
Less than 5	220 (69.62)	17 (5.38)	42 (13.29)	37 (11.71)	46.9423*
Five and more	1,336 (52.58)	83 (3.27)	438 (17.24)	684 (26.92)	
Number of living children					
Less than 5	317 (68.32)	17 (3.66)	63 (13.58)	67 (14.44)	48.5848*
Five and more	1,239 (51.78)	83 (3.47)	417 (17.43)	654 (27.33)	
Contraceptive use and intention					
Using modern method	18 (30.51)	0 (0.00)	16 (27.12)	52 (42.37)	37.7809*
Non-User intends to use later	142 (56.13)	4 (1.58)	24 (9.49)	83 (32.81)	
Does not intend to use	1,396 (54.85)	96 (3.77)	440 (17.29)	613 (24.09)	
Husband attends school					11.2358*
No	257 (51.71)	26 (5.23)	71 (14.29)	143 (28.77)	
Yes	1,299 (55.04)	74 (3.14)	409 (17.33)	578 (24.49)	
Husband works					
No	916 (52.10)	31 (1.7)	323 (18.37)	488 (27.76)	62.3006*
Yes	640 (58.23)	69 (6.28)	157 (14.29)	233 (21.20)	
Maternal occupation					
Yes	37 (71.15)	0 (0.00)	0 (0.00)	15 (28.85)	13.7350*
No	1,519 (54.15)	100 (3.57)	480 (17.11)	706 (25.17)	
Mass media exposure					
No	1,234 (54.08)	76 (3.33)	414 (18.14)	558 (24.45)	16.1466*
Yes*	322 (56.00)	24 (4.17)	66 (11.48)	163 (28.35)	

^*^ *p*-value less than 0.025.

**Table 2.** Results of Multivariable Multinomial Logistic Regression for Assessing Factors Associated with Desire for More Children in Somaliland

Background characteristics	Desire for more children		
	Within 2 years vs. after 2 years	Within 2 years vs. undecided	Within 2 years vs. wants no more
	RRR (95% CI) *p*-V*	RRR (95% CI) *p*-V*	RRR (95% CI) *p*-value*
Maternal age in 5-year groups			
Ref (15–19)	1	—	—
20–24	12.850 (−9701.633, −9727.334)	−3.081 (−5.301772, −.8604388)*	−4.652 (−7.203235, −2.101578)*
25–29	12.831 (−9701.652, −9727.314)	−1.476 (−3.242704, −0.2905485)	−2.367 (−4.000641, −0.7348949)*
30–34	13.831 (−9700.652, −9728.314)	−0.999 (−2.749331, −0.7509542)	−2.246 (−3.87141, −0.6221878)*
35–39	14.370 (−9700.112, 9728.854)	−0.528 (−2.279508, 1.2217)	−1.455 (−3.07441, 0.1629339)
40–44	−2.087 (−9779.456, 9775.281)	0.616 (−1.130112, 2.363523)	−0.0798 (−1.692077, 1.532393)
45–49	15.292 (−9699.191, 9729.776)	0.944 (−0.8200995, 2.70878)	0.812 (−0.811441, 2.437192)
Region			
Ref (Awdal)	1	—	—
Woqooyi Galbeed	2.533 (0.9548539, −4.112541)*	−0.481 (−0.9702783, 0.0080957)	−1.055 (−1.448558, −0.6626782)*
Togdheer	1.843 (0.2818393, 3.40513)*	−0.167 (−0.6609333, 0.3251893)	−0.668 (−1.057154, −0.2795773)*
Sool	−0.587 (−2.315748, 1.140664)	0.391 (−0.0687962, 0.8509529)	−2.237 (−2.67008, −1.805332)*
Sanaag	1.844 (0.3267136, 3.361815)*	0.268 (−0.1998969, 0.7369822)	−1.571 (−1.982657, −1.161181)*
Residence			
Ref (Rural)	1	—	—
Urban	1.869 (1.170609, 2.568489)*	−0.331 (−0.6025332, −0.0596717)*	0.181 (−0.068082, 0.4308364)
Nomadic	0.220 (−0.5410878, 0.9822311)	0.019 (−0.3226378, 0.3620254)	−1.054 (−1.379299, −0.7301444)*
Maternal education level			
Ref (No Education)	1	—	—
Primary	0.346 (−0.7657802, 1.458437)	−0.226 (−0.7509926, 0.2982449)	1.222 (0.7831874, 1.661418)*
Secondary	3.384 (2.242141, 4.527025)*	−17.337 (−3030.024, 2995.348)	2.064 (1.383816, 2.746108)*
Higher	−17.646 (−19256.44, 19221.14)	1.500 (0.0157459, 2.985531)*	−16.644 (−7913.933, 7880.645)
Sex of household head			
Ref (Male)	1	—	—
Female	0.116 (−0.3897481, 0.6218034)	0.004 (−0.2336805, 0.2435586)	−0.0175 (−0.2450043, 0.2098102)
Wealth Index			
Ref (Lowest)	1	—	—
Second	0.848 (0.0077496, 1.689065)*	−0.665 (−1.101971, −0.2281212)*	−0.100 (−0.4703349, 0.2685147)
Middle	0.712 (−0.2802038, 1.705171)	−0.020 (−0.412255, 0.3709905)	0.595 (0.2223875, 0.9690857)*
Fourth	2.003 (1.095309, 2.911132)*	−0.408 (−0.8940271, 0.0775884)	1.111 (0.6943721, 1.529326)*
Highest	0.901 (−0.1187478, 1.922206)	−0.126 (−0.6078322, 0.354151)	0.633 (0.188926, 1.078529)*
Total children ever born			
Ref (Less than 5)	1	—	—
Five and more	−15.987 (−2505.195, 2473.219)	−0.615 (−1.265704, 0.0338043)	0.562 (−0.1447556, 1.268758)
Number of living children			
Ref (Less than 5)	1	—	—
Five and more	15.859 (−2473.348, 2505.066)	0.398 (−0.1400908, 0.9362274)	0.260 (−0.3136808, 0.8340138)
Contraceptive use and intention			
Ref (Using modern method)	1	—	—
Non-User intends to use later	14.516 (−4948.39, 4977.423)	−1.831 (−2.693451, −0.9698756)*	−0.704 (−1.504815, 0.0948462)
Does not intend to use	15.732 (−4947.174, 4978.639)	−1.819 (−2.585809, −1.0526)*	−1.784 (−2.53701, −1.032599)*
Husband attends school			
Ref (No)	1	—	—
Yes	0.559 (−0.1176321, 1.236955)	−0.208 (−0.5559896, 0.1398856)	−.153 (−0.4631419, 0.1568344)
Husband works			
Ref (No)	1	—	—
Yes	1.641 (1.069618, 2.214014)*	−0.199 (−0.4992897, 0.0994027)	−0.580 (−0.8597942, −0.3007733)*
Maternal occupation			
Ref (Yes)	1	—	—
No	16.353 (−5155.734, 5188.44)	17.210 (−2746.182, 2780.602)	0.346 (−0.421332, 1.115124)
Mass media exposure			
Ref (No)	1	—	—
Yes	−0.324 (−1.056479, −0.4069064)	−0.509 (−0.8899957, −0.1288795)*	−0.362 (−0.7085521, −0.01744)*
Constant*	−53.312 (−12126.1, −12019.48)	−15.470 (−2778.863, −2747.922)	2.104 (0.142065, −4.066631)*

^*^ *p*-value less than 0.025.

Ref, reference category; RRR, relative risk ratios; CI, confidence interval.

### Ethical consideration statement

The study was executed while upholding the highest ethical standards. It adhered to the guidelines set forth by the Helsinki Declaration of the Human Participant Research Association. Importantly, no human biological samples were utilized in this research, and no personally identifiable information was obtained.

## Results of the Study

### Prevalence of fertility desire among reproductive-age women in Somaliland

The findings of the study in Table 3 revealed that the majority of reproductive-age women in Somaliland, 54.4% (95% CI: 52.6%−56.3%), desire to have more children within the next 2 years, highlighting a strong preference for larger families. However, a significantly smaller proportion, 3.5% (95% CI: 2.8%−4.2%), want more children after 2 years, indicating a potential shift in fertility preferences over time. Also, nearly one in five women, 16.8% (95% CI: 15.4%−18.2%), are undecided about having more children, suggesting uncertainty or changing family planning intentions. Finally, a quarter, 25.2% (95% CI: 23.6%−26.8%), of women do not want any more children, reflecting diverse family planning goals and a desire for smaller families.

**Table 3.** Prevalence of Desire for More Children Among Reproductive Women in Somaliland

Desire for more children	Proportion	SE	95% CI
Wants within 2 years	0.544	0.009	0.526–0.562
Wants after 2 years	0.035	0.003	0.028–0.042
Undecided	0.168	0.006	0.154–0.182
Wants no more	0.252	0.008	0.236–0.268

SE, standard error; CI, confidence interval.

In Table 4, the sociodemographic characteristics among married reproductive age women in Somaliland reveal significant disparities across various aspects. The majority of women (88.94%) fall within the age group of 25–49 years, with the highest percentage (28.04%) observed in the 35–39 age group. This suggests a relatively low prevalence of adolescent mothers (0.39%) aged 15–19. Regionally, the distribution is relatively balanced, with the highest proportion (27.02%) residing in Sool, followed by Woqooyi Galbeed (22.75%). In terms of residence, a slight majority (35.39%) live in rural areas, while a considerable portion (35.14%) live in urban settings. Nomadic women constitute a significant proportion (29.47%) of the population. Also, the data reveal a significant educational gap among women, with the majority (89.53%) having no formal education. Only a small percentage (7.32%) have attained primary education, while those with secondary or higher education are even fewer (2.66% and 0.49%), respectively. Furthermore, most households (62.97%) are headed by men, reflecting traditional gender roles.

**Table 4.** Sociodemographic Characteristics Among Married Reproductive Age Women in Somaliland

Background characteristics	Categories	Frequency (*n*)	Percentage (%)
Maternal age in 5-year groups	15–19	11	0.39
	20–24	100	3.50
	25–29	353	12.36
	30–34	578	20.23
	35–39	801	28.04
	40–44	620	21.70
	45–49	394	13.79
Region	Awdal	297	10.40
	Woqooyi Galbeed	650	22.75
	Togdheer	572	20.02
	Sool	772	27.02
	Sanaag	566	19.81
Residence	Rural	1,011	35.39
	Urban	1,004	35.14
	Nomadic	842	29.47
Maternal education level	No Education	2,558	89.53
	Primary	209	7.32
	Secondary	76	2.66
	Higher	14	0.49
Sex of household head	Male	1,799	62.97
	Female	1,058	37.03
Wealth Index	Lowest	1,104	38.64
	Second	356	12.46
	Middle	469	16.42
	Fourth	387	13.55
	Highest	541	18.94
Total number of children ever born	Less than 5	316	11.06
	Five and more	2,541	88.94
Number of living children	Less than 5	464	16.24
	Five and more	2,393	83.76
Contraceptive use and intention	Using modern method	59	2.07
	Non-User intends to use later	253	8.86
	Does not intend to use	2,545	89.08
Husband attends school	No	497	17.40
	Yes	2,360	82.60
Husband works	No	1,758	61.53
	Yes	1,099	38.47
Maternal occupation	Yes	52	1.82
	No	2,805	98.18
Mass media exposure	No	2,282	79.87
	Yes	575	20.13

The wealth distribution across categories exhibits notable disparities, with the “Lowest” wealth index category constituting the largest proportion (38.64%), indicative of substantial poverty levels. Fertility rates are remarkably high, as evidenced by the majority of women (88.94%) having borne five or more children, with a comparable percentage (83.76%) having five or more living offspring. Contraceptive use remains limited, with an overwhelming majority (89.08%) not intending to utilize such methods and a mere fraction (2.07%) currently employing modern contraceptive techniques. Although a significant proportion of husbands (82.60%) have received formal education, a majority (61.53%) lack formal occupations. This stands in stark contrast to the severely limited employment opportunities for women, with only a minute percentage (1.82%) currently engaged in formal employment. Additionally, the majority of women (79.87%) have restricted access to mass media, suggesting minimal exposure to information pertaining to reproductive health and related subjects. These findings underscore the critical need for implementing targeted interventions to enhance access to education, health care, and family planning services, particularly for women in rural areas and individuals from lower socioeconomic strata.

The bivariate analysis explores the association between various background characteristics and the desire for more children among reproductive age women in Somaliland. The results, as presented in Table 1, reveal several significant associations. For instance, a significant association was found between maternal age and the desire for more children (χ^2^ = 647.9734, *p* < 0.001). This means that younger women (15–19) were more likely to desire more children within 2 years, while older women (40–49) were more likely to want no more children. This trend likely reflects the influence of cultural norms and the changing life circumstances of women as they age. In terms of residence and fertility desires, results indicate that women residing in rural areas were more likely to desire more children than those in urban or nomadic settings (χ^2^ = 70.7427, *p* < 0.001). This suggests that rural women may face different social and economic pressures that influence their fertility desires.

The study on Somaliland women’s education and fertility preferences reveal a significant correlation between educational attainment and desired number of children (χ^2^ = 102.4772, *p* < 0.001). Women without formal education were found to have a greater inclination toward larger families compared to their more educated counterparts. This relationship suggests that education may influence fertility preferences by enhancing knowledge of family planning options and enabling women to make well-informed decisions. Regarding household leadership and fertility desires in Somaliland, the research indicates that women in male-headed households exhibited a stronger preference for more children than those in female-headed households (χ^2^ = 11.5366, *p* < 0.05). This disparity could be attributed to conventional gender roles and societal expectations surrounding childbearing. Additionally, the study identified a notable link between wealth status and fertility aspirations among married women of reproductive age in Somaliland (χ^2^ = 62.0740, *p* < 0.001). Women from lower economic backgrounds demonstrated a higher likelihood of wanting more children compared to those from wealthier households. This finding underscores the impact of socioeconomic factors on fertility preferences, possibly due to restricted access to resources and opportunities among less affluent women.

In addition, the results obtained from the parity and fertility desires show that both the total number of children ever born and the number of living children significantly influenced fertility desires (χ^2^ = 46.9423 and 48.5848, respectively, *p* < 0.001). Women with fewer children were more likely to desire more children. This finding suggests that parity plays a role in shaping fertility desires. In terms of contraceptive use with intention and fertility desires, Somaliland women who were not currently using contraception but intended to use it later were more likely to desire more children compared to those who did not intend to use contraception (χ^2^ = 37.7809, *p* < 0.001). This finding highlights the influence of contraceptive attitudes on fertility desires. For maternal occupation and fertility desires, Somaliland women who were currently employed were less likely to desire more children compared to those who were not (χ^2^ = 13.7350, *p* < 0.05). This suggests that employment opportunities may impact women’s fertility desires, potentially by increasing their economic independence and changing their life priorities. Also, results obtained from analysis show that a significant association was observed between mass media exposure and fertility desires (χ^2^ = 16.1466, *p* < 0.05). Women who had access to mass media were slightly less likely to desire more children compared to those who did not. This suggests that exposure to information and media influence might be a factor in shaping fertility desires. Therefore, the findings reveal the intricate dynamics of multiple socioeconomic, cultural, and personal influences that affect fertility intentions among married women of reproductive age in Somaliland.

This multivariable multinomial logistic regression analysis in Table 2 sheds light on factors associated with the desire for more children in Somaliland, examining women’s fertility decisions across four categories: wanting more children within 2 years, after 2 years, being undecided, and wanting no more children. For instance, the analysis reveals a strong association between maternal age and the desire for more children within 2 years. Women aged 20–24 and older are significantly less likely to desire more children within 2 years compared to women aged 15–19. This pattern holds true across all comparison groups, suggesting that older women are less likely to want more children in the immediate future or at all. For example, women aged 20–24 had an RRR of −3.081 (95% CI: −5.302, −0.860, *p* < 0.05) for desiring more children within 2 years compared to after 2 years. Similarly, women aged 25–29 had an RRR of −1.476 (95% CI: −3.243, −0.291, *p* < 0.05) for the same comparison. Region also plays a role in shaping women’s childbearing desires. Compared to women from Awdal, women from Woqooyi Galbeed, Togdheer, Sool, and Sanaag are significantly less likely to desire more children within 2 years. This suggests regional variations in family planning attitudes and practices, potentially influenced by socio-economic factors, cultural norms, or access to family planning services. For instance, women from Woqooyi Galbeed had an RRR of −0.481 (95% CI: −0.970, 0.008, *p* < 0.05) for desiring more children within 2 years compared to after 2 years.

Furthermore, urban residence is associated with a greater desire for more children within 2 years compared to rural residence. However, this difference disappears when considering longer-term desires or uncertainty about wanting more children. This suggests that while urban women may be more likely to want more children in the near future, their long-term family planning aspirations might align more closely with those of rural women. For urban women, the RRR for desiring more children within 2 years compared to after 2 years was −0.331 (95% CI: −0.603, −0.060, *p* < 0.05). Education level also emerges as a significant factor. Women with higher education levels are significantly more likely to desire more children within 2 years compared to those with no education. This association remains consistent across all comparison groups, potentially highlighting the influence of education on women’s empowerment and decision-making regarding family planning. However, there is a significant decline in the desire for more children within 2 years for women with secondary education. This unexpected pattern warrants further investigation. For women with secondary education, the RRR for desiring more children within 2 years compared to wanting no more children was 2.064 (95% CI: 1.384, 2.746, *p* < 0.05).

The research findings indicate a significant inverse relationship between exposure to mass media and the desire for additional children within a 2-year timeframe among women. This observation suggests that mass media campaigns focused on family planning may effectively influence attitudes and contribute to a reduction in desired family size. The study reported an RRR of −0.362 (95% CI: −0.709, −0.017, *p* < 0.05) for women exposed to mass media desiring more children within 2 years compared to being undecided. Interestingly, several other factors, including household head gender, wealth index, total children ever born, number of living children, and spousal education or employment status, did not demonstrate significant influence on the desire for more children within 2 years. Nevertheless, the research identified a notable association between contraceptive use intention and the desire for additional children within a 2-year period, emphasizing the critical role of family planning accessibility and awareness. For women intending to use contraception at a later time, the RRR for desiring more children within 2 years compared to wanting no more children was −0.704 (95% CI: −1.505, 0.095, *p* < 0.05). These results highlight the intricate interplay of socio-economic, cultural, and individual factors shaping women’s reproductive desires in Somaliland. To develop targeted and effective family planning interventions, further investigation is required to elucidate these associations and address the specific needs and contexts of the population.

## Discussion

This study investigated the prevalence and determinants of fertility decisions among married reproductive-age women in Somaliland using data from the 2020 SLHDS. Our findings reveal a complex interplay of individual, social, and economic factors influencing women’s fertility intentions, underscoring the need for tailored interventions to address the complex dynamics of family planning in the region.

The study revealed a strong preference for larger families in Somaliland, with over half (54.4%) of women desiring more children within the next 2 years. This is significantly higher than the global trend toward lower fertility rates.^1^ This finding, however, aligns with previous studies indicating a higher total fertility rate (TFR) in Somaliland, reported to be 5.7 per woman.^12^ The persistent high TFR despite the global decline suggests unique socio-economic and cultural contexts at play in Somaliland that warrant further exploration.

Our finding that younger women express a stronger desire for more children is consistent with demographic trends observed in other SSA countries where early marriage and childbearing are prevalent.^23,30^ However, the specific magnitude of this effect may vary depending on cultural context and access to education. Similarly, the association between rural residence and higher fertility desires has been documented in numerous studies,^7,23^ often attributed to limited access to family planning services and reliance on children for agricultural labor. This is further supported by the fact that Somaliland, despite recent improvements in health care, continues to face challenges regarding child mortality.^32,33^ In this context, large families can be viewed as a form of insurance against child loss, ensuring the survival of at least some offspring to provide support in old age.^34–36^

Interestingly, higher levels of education were unexpectedly associated with a higher likelihood of desiring more children within 2 years. This seemingly counterintuitive finding may be due to increased awareness of family planning options and a greater sense of control over reproductive choices among women with higher education levels. This contrasts with some studies in other African contexts that show an inverse relationship between education and fertility desires.^27,37^ However, the desire for more children within 2 years declined significantly for women with secondary education, which requires further investigation. This may indicate a shift in aspirations or economic opportunities at the secondary education level that warrants further qualitative exploration.

Our finding that exposure to mass media is associated with a lower likelihood of desiring more children aligns with studies across SSA countries.^27^ This supports the importance of mass media campaigns to educate women about family planning and other options.

Our findings must also be interpreted in the context of Somaliland’s unique socio-political and economic landscape. Unlike many other countries in SSA, Somaliland has experienced a period of relative stability and self-governance, but faces challenges related to international recognition and limited access to external aid.^38^ Furthermore, cultural and religious beliefs may play a prominent role in shaping fertility preferences, requiring culturally sensitive and tailored interventions.^35^ Somali society also has cultural norms that traditionally value large families, due to political instability and wars between clans.^39,40^ This is further supported by.^41^ As well, other potential contributing factors include economic contributions from children and religious beliefs. Therefore, a complex combination of mortality risks, cultural values, and economic considerations may contribute to the persistence of high fertility desires despite awareness of family planning.

Addressing the high fertility rate in Somaliland is crucial for achieving several SDGs, particularly SDG 3 (Good Health and Well-being), SDG 5 (Gender Equality), and SDG 8 (Decent Work and Economic Growth).^42^ By empowering women to make informed choices about family size, we can improve maternal and child health outcomes, promote gender equality, and foster sustainable economic development. This also aligns with the African Union’s Agenda 2063, which emphasizes the importance of investing in human capital and promoting sustainable development.^43^ Furthermore, the findings of this study can inform the development and implementation of Somaliland’s national health and development plans, ensuring that family planning programs are tailored to the specific needs and contexts of the population.^44^

This study contributes significantly to the understanding of fertility decisions in Somaliland by examining the role of individual-, social-, and community-level factors. The study offers valuable insights into the unique context of Somaliland, particularly the influence of socioeconomic pressures, cultural norms, and limited access to resources on fertility decisions. The findings also highlight the potential impact of education and mass media exposure on fertility desires, emphasizing the need for targeted interventions to support women in achieving their desired reproductive outcomes.

The strengths of this study include the use of nationally representative data from the 2020 SLHDS, which allows for generalizable findings. The use of multinomial logistic regression also allowed us to examine the factors associated with different categories of fertility desires. However, this study has limitations that should be considered when interpreting the findings. The cross-sectional design does not allow for causal inferences about the relationship between factors and fertility decisions. The study also relied on self-reported data, which may be subject to recall bias. Additionally, the study focused on married women of reproductive age, excluding unmarried women and individuals outside the 15–49 age range, potentially limiting the generalizability of the findings. Furthermore, the SLHDS data, while comprehensive, may not capture the nuances of cultural and religious beliefs that influence fertility decisions.

## Conclusions

This study offers a valuable contribution to understanding the prevalence and determinants of fertility decisions in Somaliland. By revealing the complex interplay of individual, social, and economic factors shaping fertility intentions, the study highlights the importance of addressing several key factors, including education, access to family planning services, and mass media exposure. This knowledge can guide the development of effective family planning interventions tailored to the specific needs and contexts of the population, empowering women to make informed choices regarding family size and achieve their desired reproductive outcomes.

## Future Work Directions

This research prompts further research on several fronts. Conducting in-depth qualitative studies exploring the nuanced reasons behind fertility decisions, including cultural norms, religious beliefs, and individual aspirations, can provide a deeper understanding of the complexities of family planning in the region. Longitudinal studies tracking women’s fertility intentions over time can shed light on how individual and social factors shape fertility desires and how these desires translate into reproductive behaviors. Evaluating the effectiveness of existing family planning interventions in Somaliland and exploring ways to tailor these interventions to specific needs and contexts can guide program development and resource allocation.

## Data Availability

The study utilized publicly available secondary data, which can be accessed at no cost through the website https://microdata.nbs.gov.so/index.php/catalog/50.

## Abbreviations Used

EA: enumeration area
GIS: geographic information system
PSU: primary sampling units
SLHDS: Somaliland Health and Demographic Survey
TNS: temporary nomadic settlements
